# Acute Vascular Response to Hemodialysis as Measured by Serum Syndecan-1 and Endothelin-1 Levels as Well as Vascular Stiffness

**DOI:** 10.3390/jcm12237384

**Published:** 2023-11-29

**Authors:** Balázs Sági, Szilárd Kun, Rita Klaudia Jakabfi-Csepregi, Endre Sulyok, Botond Csiky

**Affiliations:** 1 2nd Department of Internal Medicine and Nephrology, Diabetes Center, Clinical Center, Medical School, University of Pécs, 7624 Pécs, Hungary; balazs.sagidr28@gmail.com (B.S.);; 2 Fresenius Medical Care Dialysis Centers, 7624 Pécs, Hungary; 3 Institute of Laboratory Medicine, Medical School, University of Pécs, 7624 Pécs, Hungary; ritacsepregi93@gmail.com; 4 Doctoral School of Health Sciences, University of Pécs, 7624 Pécs, Hungary; esulyok@t-online.hu

**Keywords:** end-stage kidney disease, hemodialysis, endothelial glycocalyx, syndecan, endothelin, arterial stiffness

## Abstract

Background: Chronic hemodialysis (HD) patients have a very high cardiovascular risk. Acute vascular changes during dialysis mediated by factors of the endothelium may have a crucial role in this. The aim of this article is to study the acute vascular changes during HD. Methods: In 29 consecutive chronic HD patients (age: 65.6 ± 10.4 years), their pre-, mid-, and post-HD plasma syndecan-1 (SDC-1) and endothelin-1 (ET-1) levels were measured. Applanation tonometry was performed before HD. Results: Their SDC-1 levels increased during HD (*p* = 0.004). Males had higher ET-1 levels. The patients were divided into two groups based on their pre-HD pulse wave velocity (PWV): PWV ≥ 12 m/s and PWV < 12 m/s. The pre-HD and mid-HD SDC-1 levels were higher in the group with a PWV ≥ 12 m/s (10.174 ± 2.568 vs. 7.928 ± 1.794 ng/mL, *p* = 0.013, and 10.319 ± 3.482 vs. 8.248 ± 1.793 ng/mL, *p* = 0.044, respectively). The post-HD ET-1 levels were higher in the patient group with a PWV ≥ 12 m/s (10.88 ± 3.00 vs. 8.05 ± 3.48 pg/l, *p* = 0.027). Patients with a PWV ≥ 12 m/s had higher pre-HD peripheral and aortic systolic blood pressures (*p* < 0.05). The total cholesterol correlated with the SDC-1 decrease during HD (r = 0.539; *p* = 0.008). The pre-, mid-, and post-HD SDC-1 correlated with ultrafiltration (r = 0.432, *p* = 0.019; r = 0.377, *p* = 0.044; and r = 0.401, *p* = 0.012, respectively). Conclusion: SDC-1 and ET-1 contribute to the vascular changes observed during HD, and they have correlations with some cardiovascular risk factors.

## 1. Introduction

The incidence and prevalence of chronic kidney disease (CKD) are increasing; the number of patients may be around 160 million worldwide [1,2,3]. Cardiovascular (CV) diseases are the most common causes of morbidity and mortality in these patients. Traditional risk factors (hypertension, diabetes, dyslipidemia, obesity, and smoking) and nontraditional risk factors (volume overload, anemia, calcium phosphate metabolism disorders, hyperkalemia, and chronic inflammation) are present [4,5,6]. End-stage kidney disease (ESKD) patients have a considerably higher risk of CV death than the general population [7,8]. In these patients, oxidative stress contributes to the development of vascular dysfunction, coronary artery disease [9], and increased arterial stiffness [10], which seems to be aggravated by the bone–vascular interaction [11,12].

The functional integrity of the endothelium can be compromised, having a crucial role in the dysfunction of the vascular endothelium, leading to acute and chronic hemodynamic changes and also to the development of hypertension and other cardiovascular disorders in CKD patients [13].

The salt- and volume-dependent hypertension of chronic HD patients and the hypotension developing as a result of ultrafiltration are much-studied topics, but their pathophysiology is not completely understood. It has been proven that both hypertension and post-dialysis hypotension have a negative effect on the survival of patients [14]. These blood pressure changes increase the shear stress applied to the surface of the endothelial cells.

The endothelial glycocalyx (GCX), a carbohydrate-rich mesh of large anionic polymers, lines the luminal side of the endothelium. It is a highly hydrated, negatively charged “firewall” that protects the endothelium from pathogenic insults. These negatively charged macromolecules can bind and reversibly store sodium, providing a first-line barrier against sodium overload in the endothelial cells [15]. Its components are glycoproteins, proteoglycans, and glycosaminoglycans [16]. The thickness and structure of the GCX depend largely on the shear stress applied to the surface of the endothelial cells. The GCX has a dynamic relationship with the circulating plasma; endothelial cells regulate its thickness and composition in response to changes in the local microenvironment. It has a crucial role in the maintenance of vascular permeability and hemostasis, has anti-inflammatory and antiatherogenic properties, and is a key mediator of flow-dependent nitric oxide (NO) synthesis. Its functional integrity can be compromised by hypercholesterolemia, diabetes mellitus, hypervolemia, vascular surgery, sepsis, hyperglycemia, and chronic kidney failure [17,18].

Plasma syndecan-1 (SDC-1) is a heparan sulfate proteoglycan expressed in endothelial cells and the main marker of endothelial GCX degradation. An elevated serum level of SDC-1 is associated with endothelial injury and can be used to characterize GCX damage. SDC-1 plays an important role in cell migration, differentiation, and proliferation. It protects the endothelial GCX from harmful effects and contributes to the attachment of platelets, leukocytes, and inflammatory cells on the surface of the endothelium. SDC-1 is a promising biomarker for the diagnosis and prognosis of vascular diseases [19,20].

The role of endothelin-1 (ET-1) in the progression of chronic kidney disease and the development of hypertension is relatively well known. ET-1 is a peptide hormone produced and released by endothelial cells. It acts on various tissues and cells and is involved in several physiological processes. As it is also a potent vasoconstrictor, it increases one’s blood pressure. It may play a role in CV diseases such as hypertension, heart attacks, and strokes. ET-1 plays an important role in cell migration, cell division, the regulation of cell division, the regulation of cell functions, and the maintenance of cell composition. However, its effects seem to be sensitive to changes in the extracellular environment and the physiological state of the cells [21,22].

We have relatively little data on the injury of the GCX in hemodialysis patients and on the role of SDC-1 and ET-1 in acute hemodynamic changes during hemodialysis treatment elicited mainly by the ultrafiltration process. Therefore, particular attention was paid to estimating the importance of GCX in HD-related acute hemodynamic changes in patients in regular HD.

## 2. The Aim of This Study

The purpose of this study was to measure the SDC-1 and ET-1 levels before, during, and after HD to clarify their role in the acute hemodynamic changes and to find a correlation between the SDC-1 and ET-1 levels, central and peripheral BP, and the CV risk factors.

## 3. Patients and Methods

This study was designed to assess the time course of vascular responses to HD sessions by measuring SDC-1, ET-1, and PWV, exploring the interrelationships of these indices of vascular functions and revealing their association with some clinical and laboratory parameters.

## 4. Patients

We selected 29 consecutive chronic HD patients from 125 patients, who participated in this prospective, cross-sectional descriptive study, conducted at the Fresenius Medical Care Dialysis Centers of Pécs. One patient withdrew his informed consent. Clinically stable chronic HD patients who had been in the dialysis program for at least 3 months were included. The eligibility criteria included an age of >18 years old, no malignant disease, no acute or chronic infection, and a stable clinical status.

Patients with lower-extremity amputations, any acute infection, malignancies, acute myocardial infarction, pulmonary edema, or hemodynamic instability were excluded from this study.

The underlying renal pathologies that progressed to ESKD included the following: diabetic nephropathy (24%), nephrosclerosis (26%), chronic glomerulonephritis (15%), polycystic kidney disease (10%), chronic interstitial nephritis (10%), renovascular disease (2%), and other/unknown causes (13%). Most of the patients (85%) received antihypertensive therapy.

The patients underwent three HD sessions per week, each lasting for 4 h. Online hemodiafiltration was carried out using Fresenius 5008 B (Fresenius Medical Care Gmbh, Bad Homburg, Germany) equipment with Helixone/Fresenius polysulfone high-flux dialyzer membranes. Their body mass index (BMI) was calculated.

**Figure 1:**
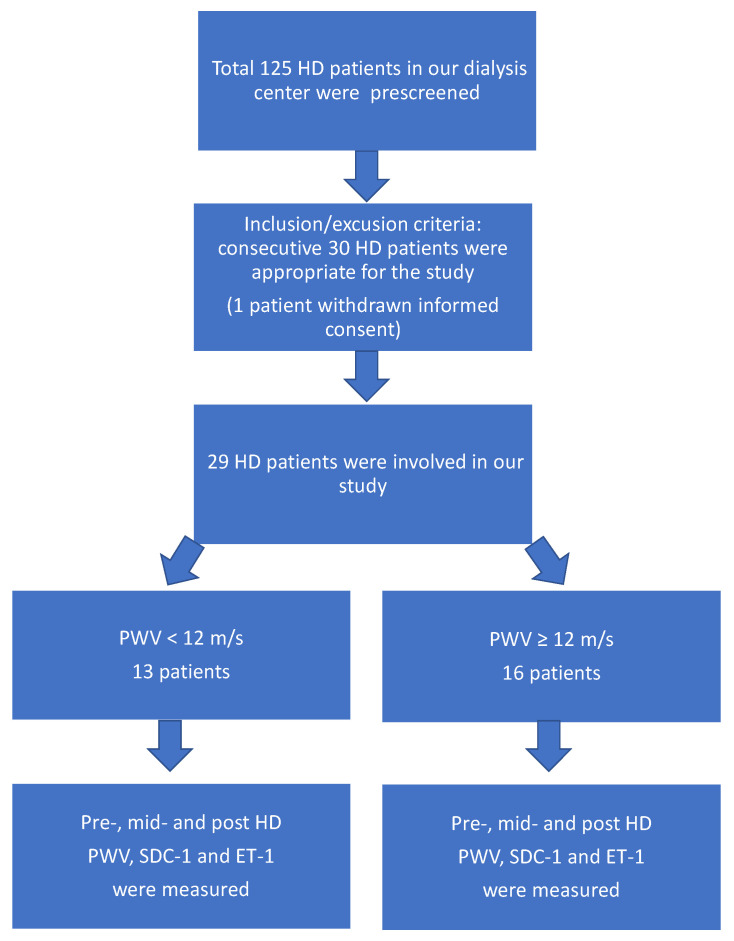
Patient-recruitment flow chart.

The patients were divided into two groups based on their pre-HD pulse wave velocity (PWV < 12 m/s vs. PWV ≥ 12 m/s), as a PWV < 12 m/s is considered normal by the ESC/ESH guidelines [23] (Figure 1).

## 5. Blood Pressure and Pulse Wave Velocity Measurements

Their BP was measured with a calibrated automated device (Fresenius 5008S (Fresenius Medical Care Gmbh, Bad Homburg, Germany) integrated blood pressure module) using an appropriate cuff size. Their carotid–femoral pulse wave velocity (cfPWV) and augmentation index (AIx) were measured via applanation tonometry (SphygmoCor System, AtCor Medical, Sydney, Australia) before the HD treatment. Pulse wave recordings were consecutively performed at two different arterial palpation locations (carotid and femoral segments). All recorded values conformed to the quality control standards incorporated in the software package provided by the manufacturer. All measurements were performed by the same operator.

## 6. Laboratory Measurements

Routine biochemical parameters were measured via the standard methods. Serum ET-1 and SDC-1 were measured via human enzyme-linked immunoassay (ELISA) kits (Sigma-Aldrich Chemie GmbH, Taufkirchen, Germany) in samples obtained pre-, mid-, and post-HD.

## 7. Patients’ Fluid Overload Assessment

We measured all the patients’ fluid overload statuses via a bioimpedance Body Composition Monitor (BCM, Fresenius Medical Care, Bad Homburg, Germany) before the hemodialysis session. Overhydration (OH) and overhydration extracellular water (OH ECW) parameters were used to assess the patients’ volume statuses.

## 8. Statistical Analysis

The results are presented with a frequency and average confidence range. The significance level was determined at *p* < 0.05. The normality of the data was determined using the Kolmogorov–Smirnov test. Correlations between continuous variables were determined by calculating linear regression using the Pearson test. The data were presented as the mean ± SD in cases of a normal distribution and as the median (lower or upper quartile) in cases of a nonnormal distribution. Trend analysis was performed using Wilcoxon’s methods. A univariate analysis of variance was used to investigate the potential effect of covariates in a model. A backward multiple regression analysis was performed to determine the relative influence of the selected independent variables on the variance of the dependent variable in the constructed model. Statistical analyses were performed using SPSS (SPSS, Inc., Chicago, IL, USA) version 21.0 software.

## 9. Ethical Consideration

This research was approved by the Local and Regional Ethical Committee (9537-PTE2023). The investigation conformed to the principles outlined in the World Medical Association Declaration of Helsinki. Informed written consent was obtained from all the participants.

## 10. Results

The clinical and baseline laboratory data of the patients are presented in Table 1.

**Figure 2:**
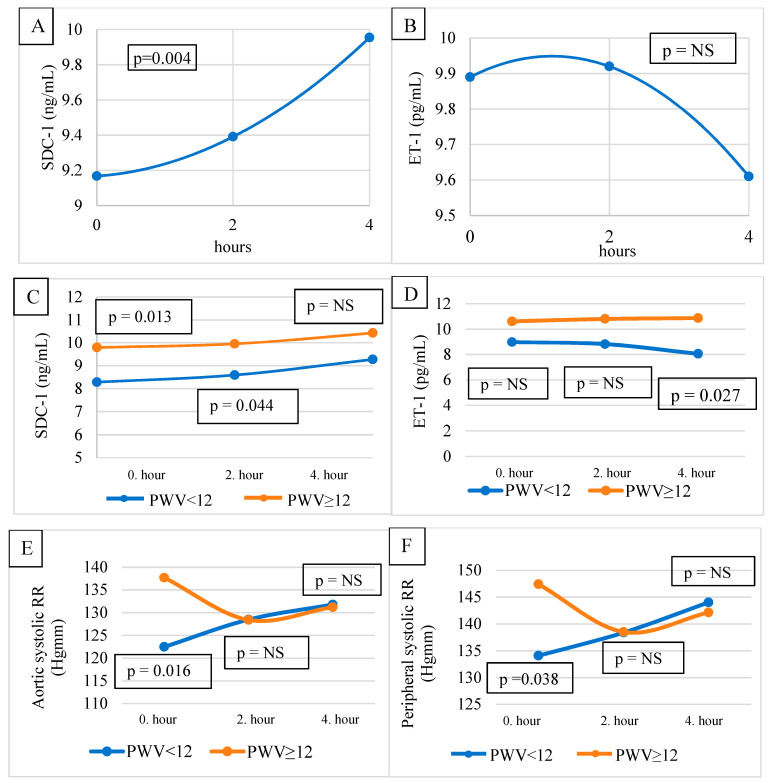
SDC-1 (**A**) and ET-1 (**B**) in 29 HD patients. SDC-1 in patients with lower and higher PVW (**C**). ET-1 in patients with lower and higher PVW (**D**). Aortic (**E**) and peripheral (**F**) BP of patients with lower or higher PVW. SDC-1, syndecan-1; ET-1, endothelin-1; NS: Not significant.

The hemodynamic parameters, SDC-1, and ET-1 levels are presented in Table 2. No significant difference was found between the SDC-1 levels pre-, mid-, and post-HD. Similarly, no significant difference was observed between the ET-1 values measured before, during, and after HD. On the other hand, according to Wilcoxon’s trend analysis, the change in SDC-1 pre-HD, mid-HD, and post-HD showed a significantly increasing trend (*p* = 0.004) (Figure 2A). A decreasing trend for ET-1 was observed during the observation period (Figure 2B), which was not significant.

The pre-HD SDC-1 level in men (10.547 ng/mL) was significantly (*p* = 0.001) higher than that in women (7.689 ng/mL, *p* = 0.001). Similarly, the mid-HD and post-HD SDC-1 levels were also significantly higher in men than in women.

The patients were divided into two groups based on their pulse wave velocity (PWV < 12 m/s vs. PWV ≥ 12 m/s). No significant difference was observed in the baseline parameters of the two patient groups, except for their age and hemoglobin level (Table 1). The group 2 patients had a higher pre-HD peripheral systolic BP and central (aortic) BP than the group 1 patients. The mid-HD peripheral and central BPs were not different between the groups.

The pre-HD and mid-HD SDC-1 were higher in the PWV ≥ 12 m/s group (10.174 ± 2.568 vs. 7.928 ± 1.794 ng/mL, *p* = 0.013, and 10.319 ± 3.482 vs. 8.248 ± 1.793 ng/mL, *p* = 0.044, respectively). There was no difference in the post-HD SDC-1 levels between the groups (Table 2 and Figure 2C).

Post-HD ET-1 was higher in the PWV ≥ 12 m/s group (10.88 ± 3.00 vs. 8.05 ± 3.48 pg/mL, *p* = 0.027). There was no significant difference in the pre-HD and mid-HD ET-1 levels between the groups (Table 2 and Figure 2D).

The pre-HD peripheral (Figure 2F) and central/aortic BPs (Figure 2E) were significantly higher in the PWV ≥ 12 m/s group. The mid- and post-HD BPs were not different between the groups (Table 2).

A univariate analysis of variance was used to investigate the potential effect of age, peripheral systolic blood pressure, and Hb level on the differences in the SDC-1 and ET-1 levels between the two groups (PWV < 12 and PWV ≥ 12). No effect of the above-mentioned covariates could be detected.

**Figure 3:**
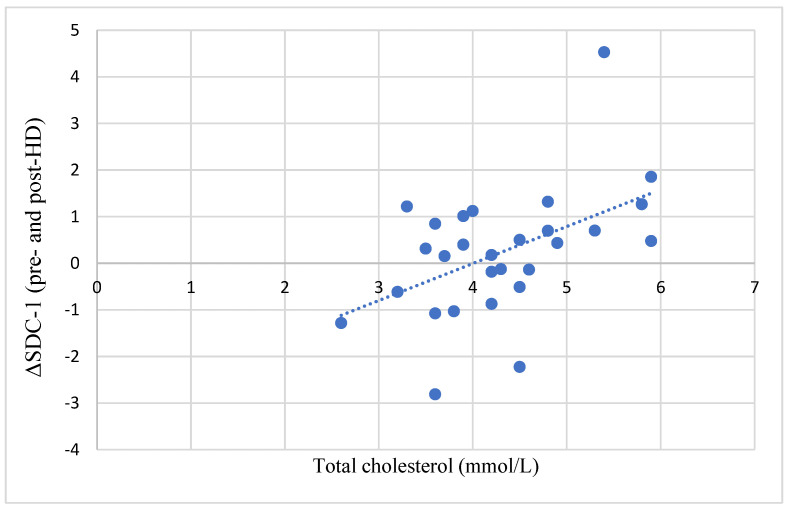
Correlation between the difference in pre- and post-HD SDC-1 and serum cholesterol levels. ΔSDC-1, syndecan-1 change; SDC-1, syndecan-1; ET-1, endothelin-1.

A significant positive correlation was found between the difference in the pre-HD and post-HD SDC-1 levels (ΔSDC-1) and the serum total cholesterol levels (Figure 3).

**Figure 4:**
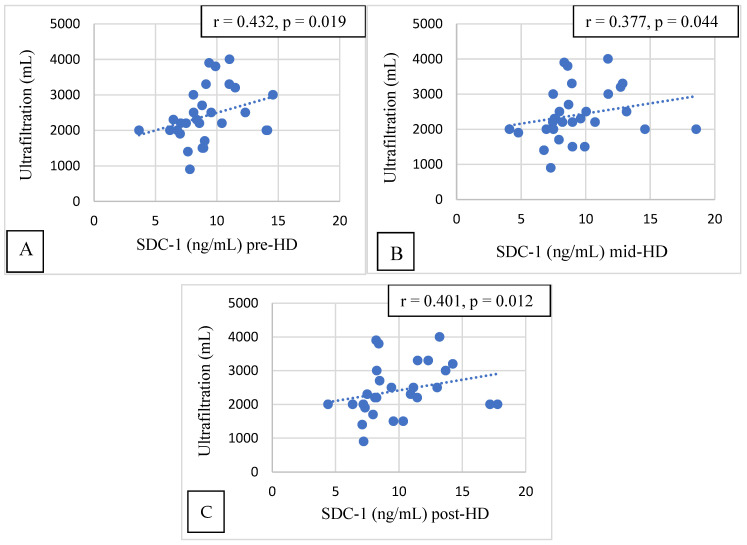
Correlation between SDC-1 measured pre-HD (**A**), mid-HD (**B**), and post-HD (**C**) and ultrafiltration volumes.

Pre-, mid-, and post-HD SDC-1 had a significant correlation with the ultrafiltration volume (*p* = 0.019, *p* = 0.044, and *p* = 0.012, respectively, Figure 4).

## 11. Discussion

We observed increasing SDC-1 levels during the HD sessions in our study. As a trend, this increase was significant. According to the literature, high SDC-1 levels may be a sign of vascular injury caused by uremic toxins, chronic inflammation [24], oxidative stress [25], volume overload [26], and endothelial dysfunction [27]. Besides the long-term effects of these factors, as the GCX serves as a mechanosensor for shear stress [28], we assume that the short-term increase in the SDC-1 levels during the HD sessions observed in our study was caused mainly by the increased shear stress applied to the endothelium. Endothelial cells also have a repair response to tissue damage. This response depends on the intrinsic regenerative capacity of the endothelial cells and the stem cells [29], the latter being abundant in patients with renal failure [30]. However, SDC-1 is also considered a marker of tissue regeneration [31]. According to this, the rising SDC-1 levels during the HD sessions might also be a sign of active tissue reparation and could be considered a beneficial adaptive response to the injury of the endothelial glycocalyx caused by increased shear stress.

We found significantly higher SDC-1 levels in men than in women (pre-, mid-, and post-HD), confirming the literature data [32,33,34], probably caused by differences in sex hormones and dietary habits.

We observed a nonsignificantly decreasing trend in ET-1 during dialysis. ET-1 is a potent vasoconstrictor, with elevated levels in HD patients. ET-1 has been described to increase during HD sessions in patients with dialysis-induced hypertension, to decrease in patients with dialysis-induced hypotension, and to be unchanged in patients whose BP did not change considerably during HD [32]. In our patients, their BP did not change considerably during dialysis, and, in line with the literature data, we did not detect a significant change in their ET-1 levels. ET-1 was probably not the relevant factor in maintaining or increasing the BP of our patients during their HD sessions. However, it might have a role in increasing the shear stress of the endothelium.

The GCX is involved in the mediation of shear-induced nitric oxide releases. Excessive nitric oxide generation is involved in HD-associated hypotension [35], as the endothelial GCX is an important regulator of vascular tone. SDC-1 is a marker of GCX damage or, eventually, its repair [36,37,38,39]. Accordingly, we assumed we would find a relationship between the SDC-1 change during dialysis and the vascular stiffness assessed via application tonometry (PWV, Aix). We could not demonstrate such an association. On the other hand, we found a close negative relationship between PWV and the change in ET-1 during dialysis, demonstrating the importance of ET-1 in regulating vascular tone during dialysis sessions.

Hypercholesterolemia is known to compromise the integrity of the GCX [16,17,26]. Greater vascular damage is assumed to be reflected by higher SDC-1 levels. In line with these data, we found a close positive correlation between the difference between pre-HD and post-HD SDC-1 and the total cholesterol of the patients (Figure 2).

Convincing evidence has been provided for the role of hypervolemia in compromising the integrity of the vascular GCX [16,17]. In HD patients, the excess extracellular fluid is removed via ultrafiltration during HD sessions. If the extracellular volume is high, more ultrafiltration is needed during HD. In our study, the pre-HD, post-HD, and mid-HD SDC-1 levels were significantly positively correlated with the ultrafiltration volume. High ultrafiltration volumes cause increased shear stress on the arterial walls. A GCX injury is a consequence of this increased shear stress, and increased SDC-1 is a marker of a GCX injury. HD patients with a high interdialytic weight gain who need high ultrafiltration volumes during HD sessions have a worse survival than patients with a lower interdialytic weight gain [40]. Several studies have revealed that prolonged HD is associated with better BP and fluid management [37,41,42]. Rapid fluid removal, which is needed when the ultrafiltration volume is high, may be associated with a higher risk of CV events and death [43]. As a consequence of a high ultrafiltration volume, intradialytic hypotension develops frequently, which is associated with increased mortality [44,45].

According to the literature data, elevated SDC-1 is associated with decreased survival [46,47]. SDC-1 elevation may be time-dependent: early SDC-1 elevation could be a sign of endothelial injury, and later elevation may be observed during the reparative process of the endothelium [24]. Substantial endothelial damage may be associated with worse survival, and on the other hand, outstanding repair capabilities could be associated with better survival [48]. As our study was cross-sectional, we could not study the effect of SDC-1 on the survival of our patients. The worse life expectancy of HD patients who need high ultrafiltration volumes may be explained in part by their endothelial GCX injury [39].

In our study, we divided our patients into two groups based on their PWV: the patients in the PWV < 12 m/s group were considered to be normal [23], and the patients in group 2 had a PWV ≥ 12 m/s, showing stiffer vessel walls than those in the group with a lower PWV (<12 m/s). We hypothesized that the group 2 patients had higher pre-HD peripheral and central BPs (at least in part) because of their more rigid arterial walls. In stiffer arteries, the injury to the endothelial GCX was more severe than in the more elastic arteries (group 1). As a sign of the endothelial injury and, eventually, because of the intensive endothelial repair needed as a consequence of the injury, the SDC-1 levels were higher pre-HD and mid-HD in group 2 compared to group 1. We also hypothesized that, in the second part of the HD session when the BP difference disappeared between the groups, the shear stress and the endothelial injury were also decreased in the arteries of group 1 patients, so the post-HD SDC-1 levels were not different between the groups. As the BP became similar in the studied groups, the ET-1 levels (post-HD) became significantly lower in group 1 as a sign of healthier arteries and, eventually, as a sign of decreasing shear stress and the initiation of a BP decrease.

According to our results, the endothelial GCX plays an important role in the regulation of vascular tone and central and peripheral BP, and eventually, it may have a considerable effect on the cardiovascular events and survival of HD patients. In these patients, several factors compromise the integrity of the GCX. We studied the effects of hypervolemia/the ultrafiltration volume, BP, hypercholesterolemia, and vascular rigidity. Increased vascular shear stress may be an important common mechanism, and SDC-1 is a valuable marker of GCX injury and repair.

## 12. Limitations of the Study

There are certain limitations in our study. As we considered this a “pilot” study, the patient number is small. The examinations should be extended to larger groups in the future. This is just a cross-sectional study. As vascular alterations have long-term effects on clinical endpoints, their effects should also be examined in long-term studies.

## 13. Conclusions

SDC-1 and ET-1 contribute to the acute vascular changes observed during HD, and they correlate with the cardiovascular risk factors. It is important to restore the integrity of the GCX to decrease the very high cardiovascular risk of these patients. Further studies are needed to clarify further factors affecting vascular tone and the vascular GCX.

## Figures and Tables

**Table 1:** Baseline clinical data.

	All Patients (n = 29)	Group 1 (PWV < 12 m/s) (n = 13)	Group 2 (PWV ≥ 12 m/s) (n = 16)	*p* *
Man/woman (n%)	15/14 (51.7/48.3)	6/7 (46.2/53.8)	9/7 (56.3/43.7)	0.135
Age (year)	65.6 ± 10.6	60.1 ± 10.8	70.2 ± 8.2	0.008
HT (n/%)	29.0 (100)	13.0 (100)	16.0 (100)	0.546
DM (n/%)	10.0 (34.4)	3.0 (23)	7.0 (43.7)	0.091
BMI (kg/m^2^)	27.4 ± 5.6	27.5 ± 6.6	27.3 ± 4.9	0.456
Hb (g/L)	110.2 ± 11.2	104.69 ± 8.7	114.7 ± 11.2	0.014
se Ca (mmol/L	2.2 ± 0.1	2.19 ± 0.1	2.2 ± 0.1	0.916
se P (mmol/L)	1.7 ± 0.5	1.8 ± 0.5	1.6 ± 0.4	0.395
se PTH (pmol/L)	35.9 ± 15.3	35.0 ± 17.9	36.8 ± 13.3	0.763
Glucose (mmol/L)	6.4 ± 1.7	6.4 ± 1.4	6.4 ± 1.7	0.970
Cholesterol (mmol/L)	4.3 ± 0.8	4.3 ± 0.7	4.2 ± 0.9	0.556
Triglyceride (mmol/L)	1.5 ± 0.6	1.7 ± 0.7	1.4 ± 0.4	0.294
HDL cholesterol (mmol/L)	1.2 ± 0.4	1.3 ± 0.5	1.1 ± 0.2	0.145
CRP (mg/L)	4.4 ± 4.3	4.1 ± 3.8	4.6 ± 4.7	0.766
Fe (µmol/L)	12.3 ± 3.8	12.6 ± 3.4	12.1 ± 4.2	0.692
Ferritin (mg/L)	311.9 ± 252.9	268.8 ± 210.9	347.0 ± 284.4	0.418
ALP (U/L)	80.1 ± 25.7	72.8 ± 16.6	86.1 ± 30.5	0.170
Creatinine (µmol/L)	719.1 ± 191.9	725.9 ± 214.7	713.6 ± 178.5	0.867
se Albumin (g/L)	39.5 ± 3.8	39.9 ± 2.7	39.2 ± 4.4	0.317
Dialysis vintage (months)	114.0 ± 34.0	107.0 ± 26.0	116.0 ± 37.0	0.678
Ultrafiltration (mL)	2413.8 ± 766.6	2492.3 ± 820.0	2338.5 ± 741.2	0.598
OH (L)	2.3 ± 1.5	2.3 ± 1.0	2.3 ± 1.8	0.457
OH ecw (%)	12.2 ± 8.3	13.4 ± 5.2	11.7 ± 10.1	0.256

HT, hypertension; DM, diabetes mellitus; BMI, body mass index; Hb, hemoglobin; se Ca, serum calcium; se P, serum phosphorus; se PTH, serum parathormone; HDL, high-density lipoprotein; CRP, C-reactive protein; Fe, iron; ALP, alkaline phosphatase; OH, overhydration; OH ecw, overhydration extracellular water. * *p*: group 1 vs. group 2.

**Table 2:** SDC-1 vales, ET-1 values, and hemodynamic parameters of HD patients.

	All HD Patients (n = 29)	Group 1 (PWV < 12 m/s) (n = 13)	Group 2 (PWV ≥ 12 m/s) (n = 16)	*p*
Systolic/diastolic BP (mmHg)				
Pre-HD	141.4/73.3 ± 17.4/17.8	134.1/72.2 ± 16.6/12.8	147.4/73.3 ± 16.1/11.1	0.038
Mid-HD	138.4/71.3 ± 19.2/10.1	138.4/75.0 ± 15.9/15.9	138.5/68.8 ± 22.0/10.1	0.988
Post-HD	142.9/72.6 ± 20.2/10.0	144.0/76.6 ± 16.7/10.1	142.1/69.8 ± 23.2/9.9	0.809
Aix (%)				
Pre-HD	33.9 ± 9.2	30.0 ± 8.5	37.1 ± 8.8	0.038
Mid-HD	33.3 ± 10.9	31.8 ± 8.8	34.5 ± 12.6	0.526
Post-HD	31.3 ± 10.0	30.3 ± 10.9	32.1 ± 9.5	0.636
cfPWV (m/s)				
Pre-HD	12.4 ± 3.6	9.2 ± 2.2	14.6 ± 2.2	0.001
Mid-HD	12.63 ± 4.4	10.06 ± 2.3	14.7 ± 4.6	0.002
Post-HD	14.1 ± 4.8	12.2 ± 5.2	15.5 ± 3.8	0.01
Aorta systolic BP (mmHg)				
Pre-HD	130.9 ± 17.4	122.5 ± 14.9	137.7 ± 16.6	0.016
Mid-HD	128.4 ± 18.0	128.46 ± 14.05	128.38 ± 21.14	0.990
Post-HD	131.4 ± 20.7	131.8 ± 19.8	131.2 ± 22.0	0.942
SDC-1 (ng/mL)				
Pre-HD	9.17 ± 2.5	7.9 ± 1.8	10.2 ± 2.6	0.013
Mid-HD	9.4 ± 3.0	8.2 ± 1.8	10.3 ± 3.5	0.044
Post-HD	9.9 ± 3.2	8.9 ± 2.3	10.8 ± 3.6	0.095
ET-1 (pg/mL)				
Pre-HD	9.9 ± 3.5	9.0 ± 3.9	10.6 ± 3.0	0.212
Mid-HD	9.9 ± 3.6	8.8 ± 3.9	10.8 ± 3.3	0.150
Post-HD	9.6 ± 3.5	8.0 ± 3.5	10.9 ± 3.0	0.027

BP, blood pressure; HD, hemodialysis; Aix, augmentation index; cfPWV, carotid–femoral pulse wave velocity; SDC-1, syndecan-1; ET-1, endothelin-1.

## Data Availability

The data underlying this article cannot be shared publicly due to Hungarian regulations and the privacy of the individuals who participated in this study. The data could be shared on reasonable request to the corresponding author if accepted by the Regional Committee for Medical and Health Research Ethics and the local data protection officials.
